# Implementing a simple pharmacovigilance program to improve reporting of adverse events associated with biologic therapy in rheumatology: Preliminary results from the Calabria Biologics Pharmacovigilance Program (CBPP)

**DOI:** 10.1371/journal.pone.0205134

**Published:** 2018-10-24

**Authors:** Caterina Palleria, Luigi Iannone, Christian Leporini, Rita Citraro, Antonia Manti, Maurizio Caminiti, Pietro Gigliotti, Rosa Daniela Grembiale, Massimo L’Andolina, Giuseppe Muccari, Maria Diana Naturale, Domenico Olivo, Giuseppa Pagano Mariano, Roberta Pellegrini, Giuseppe Varcasia, Karim Abdalla, Emilio Russo, Francesco Ursini, Giovambattista De Sarro

**Affiliations:** 1 Department of Health Sciences, University of Catanzaro “Magna Graecia”, Catanzaro, Italy; 2 Associazione Calabrese per la Ricerca in Reumatologia (ACRR), Catanzaro, Italy; 3 Rheumatology Unit, Grande Ospedale Metropolitano “Bianchi-Melacrino-Morelli”, Reggio Calabria, Italy; 4 Rheumatology Outpatient Clinic, Azienda Ospedaliera Provinciale Cosenza, Cosenza, Italy; 5 Rheumatology Outpatient Clinic, Azienda Sanitaria Provinciale Vibo Valentia, Vibo Valentia, Italy; 6 Rheumatology Outpatient Clinic, Azienda Ospedaliera “Pugliese-Ciaccio”, Catanzaro, Italy; 7 Rheumatology Outpatient Clinic, Azienda Sanitaria Provinciale Crotone, Crotone, Italy; 8 Rheumatology Unit, Azienda Ospedaliera “SS Annunziata”, Cosenza, Italy; 9 Rheumatology Unit, Ospedale Castrovillari, Castrovillari, Italy; 10 Department of Medical and Surgical Sciences, University of Catanzaro “Magna Graecia”, Catanzaro, Italy; SERGAS and IDIS, SPAIN

## Abstract

**Introduction:**

Post-marketing surveillance activities (namely pharmacovigilance) are crucial to favor the early detection of unexpected adverse events (AEs) and/or serious adverse reactions (SAEs). Indeed, spontaneous reporting of AEs has been demonstrated to underestimate the number of events in different clinical settings. Aim of the present study is to report the preliminary data of a Regional (Calabria, Italy) Pharmacovigilance Program (CBPP) aimed at improving AEs’ reporting associated with biologics use in rheumatology.

**Materials and methods:**

We developed a simple, cost-effective pharmacovigilance program based on regular training sessions for physicians (stimulated reporting), periodical phone calls by a clinical pharmacologist aimed at identifying new events and stimulating self-awareness and encouraging reporting to the physician during the subsequent follow-up visit for minor AEs. To test this approach, all consecutive patients undergoing treatment with one biologic agent at eight rheumatology centers during a two-years period were invited to participate. Collected AEs were compared to the number of AEs spontaneously reported for the same molecules in the same centers before starting the protocol.

**Results:**

During the study period, 399 patients (245 females; mean age: 58 ± 11 years) were started on treatment with biologics for active RA (n = 211, 52.9%), PsA (n = 119, 29.8%) or AS (n = 69, 17.3%) at eight rheumatology centers. A total of 125 AEs (31.3%) and 9 SAEs (2.3%) were reported during the two-years study period. In the control cohort (comprising 368 consecutive patients started on treatment with bDMARDs during a two-years period before CBPP study) only 42 (11.4%) AEs and no SAEs were reported (p < 0.0001). The most common AEs were injection site reactions and skin disorders.

**Conclusions:**

In conclusion, our study provides further evidence of a critical role of active pharmacovigilance in detection, reporting and analysis of AEs in rheumatology.

## Introduction

Inflammatory arthritides is an umbrella term comprising a heterogeneous group of chronic immune-mediated diseases affecting the joints and tendons such as rheumatoid arthritis (RA), psoriatic arthritis (PsA), and ankylosing spondylitis (AS) and affecting ∼5% of population worldwide [1]. Patients with inflammatory arthritis experience recurring or chronic episodes of joints/tendons inflammation, leading ultimately to subversion of the normal anatomy and, consequently, a variable degree of functional disability [2].

Treatment options were limited for a long time to nonsteroidal anti-inflammatory drugs (NSAIDs), corticosteroids (CCs) and conventional synthetic disease-modifying anti-rheumatic drugs (csDMARDs), in particular methotrexate (MTX) [3–5].

In the last two decades, clinical practice has been revolutionized following the development and marketing of several new biologic DMARDs (bDMARDs) resulting in a dramatic improvement in the management of refractory patients [3]. These molecules can selectively target soluble mediators involved in the development and maintenance of inflammatory processes such as tumor necrosis factor-alpha (TNF-α) [6], interleukin-1 (IL-1) [7] and -6 (IL-6) [8] or surface molecules involved in T-cells activation (such as the CTLA-4—CD80/86 pathway) [9] or B cell survival signals (such surface CD20) [10]. At present, five TNF-α antagonists (TNFi) (adalimumab—ADA; etanercept—ETN; certolizumab pegol—CZP and golimumab—GOL, for subcutaneous administration; infliximab—IFX for intravenous infusion), one IL-17 inhibitor (secukinumab—SEC), one IL-12/23 inhibitor (ustekinumab—UST), one IL-6 receptor antagonist (tocilizumab—TCZ), one IL-1 inhibitor (anakinra—ANA), one T-cell co-stimulation inhibitor (abatacept—ABT), and one B-cell depleting agent (rituximab—RTX) are approved by European Medicines Agency (EMA) for treatment of patients with inflammatory arthritis. In details, all TNFis are approved for either RA, PsA or AS; SEC is approved for PsA and AS; ABT, TCZ and ANA are approved for RA only while, UST is approved for PsA.

All bDMARDs, due to their higher costs, are currently recommended for the treatment of patients with active disease and insufficient response or intolerance to csDMARDs [11,12], with the exception of individuals with definite clinical characteristics, such as those with axial-predominant spondyloarthritis (axSpA) [13], in which bDMARDs are recommended as a first-line therapy following the unsuccess of NSAIDs or topical steroid injections. Besides their well-established role in ameliorating control of disease activity, bDMARDs have been associated with safety concerns. Infections (including tuberculosis and opportunistic) [14,15], skin cancers [16], demyelinating leukoencephalopathy [17] and hepatitis B reactivation [18] have been reported during therapy with bDMARDs in clinical trials and real-life studies.

In this context, post-marketing surveillance activities (namely pharmacovigilance) are crucial to favor the early detection of unexpected adverse events (AEs) and/or serious adverse reactions (SAEs). Indeed, spontaneous reporting of AEs has been demonstrated to underestimate their number [19,20] in different clinical settings. At odds, active pharmacovigilance activities may actually improve detection and reporting of AEs [21] and represent a powerful tool to better define the safety profile of marketed molecules and to establish risk factors or frail patient phenotypes.

Aim of the present study is to report the preliminary data of a Regional (Calabria, Italy) Pharmacovigilance Program aimed at improving reporting of AEs associated with biologics use in rheumatology.

## Materials and methods

### Calabria Biologics Pharmacovigilance Program

The Calabria Biologics Pharmacovigilance Program (CBPP) is a multicenter pharmacovigilance study aimed at improving the continuous monitoring of safety of bDMARDs treatment in clinical practice. The purpose of the program is to implement a simple active AEs reporting system in all tertiary rheumatology clinics dealing with bDMARDs distributed across Calabria Region (Italy) in different fields of medicine including rheumatology, gastroenterology and dermatology. Since its starting in 2016, the program develops through regular training sessions for physicians during dedicated one-day pharmacovigilance courses (at least two courses/year) in order to improve awareness/compliance and therefore boost the reporting (stimulated reporting) attitude during scheduled follow-up visits. The physician dealing with an AE is provided with a direct interface with the Clinical Pharmacology and Pharmacovigilance Unit at University of Catanzaro “Magna Graecia” by either email, program website and dedicated phone number. Structured data are collected for each AE, including Medical Dictionary for Regulatory Activities (MedDRA) classification of the event, duration, severity and outcome. Furthermore, during the whole follow-up period, patients received periodical phone calls by a clinical pharmacologist (approximately one every three months) aimed at identifying new events, stimulating self-awareness and encouraging reporting to the physician during the subsequent follow-up visit for minor AEs. The global costs associated with the program for the rheumatology therapeutic area are related to the recruiting of one dedicated clinical pharmacologist (EUR 28.000/year) and the expenses for the organization of training courses and meetings (approximately EUR 10.000/year).

The study protocol was approved by the local Ethics Committee (Comitato Etico Regionale Calabria, Italy), protocol number 278/2015. Informed consent was obtained from all patients at the time of enrollment which were informed that medical records will be anonymously utilized for this study. All procedures were performed in accordance with the 1964 Declaration of Helsinki and its later amendments.

### Patients

In this preliminary report, we provide the data obtained during the first two years of the CBPP—Rheumatology therapeutic area. To this purpose, all consecutive patients undergoing treatment with one bDMARD at eight rheumatology centers (Rheumatology Outpatient Clinic, Azienda Ospedaliera “Pugliese-Ciaccio”, Catanzaro, Italy; Rheumatology Unit, Grande Ospedale Metropolitano “Bianchi-Melacrino-Morelli”, Reggio Calabria, Italy; Rheumatology Outpatient Clinic, Azienda Ospedaliera “Mater Domini”, Catanzaro, Italy; Rheumatology Outpatient Clinic, Azienda Ospedaliera Provinciale Crotone, Crotone, Italy; Rheumatology Unit, Azienda Ospedaliera “SS Annunziata”, Cosenza, Italy; Rheumatology Outpatient Clinic Azienda Ospedaliera Cosenza, Cosenza Italy; Rheumatology Unit, Ospedale Castrovillari, Castrovillari, Italy; Rheumatology Outpatient Clinic, Azienda Sanitaria Provinciale Vibo Valentia, Vibo Valentia, Italy) between January 1, 2016 and December 31, 2017 and satisfying predefined criteria were included. These centers account for > 90% of total bDMARDs prescription for the rheumatology area in Calabria Region.

Patients were included in the protocol if met all the following inclusion criteria:

1. age ≥ 18 years;
2. past diagnosis of RA, AS or PsA;
3. starting treatment with one bDMARD.

The date of the first bDMARD prescription during the study period represented the “index date” for each individual patient. From the index date onward, the patient was started on a hybrid reporting system, characterized by “stimulated” reporting of AEs by the managing physician during each outpatient visit and intermediate phone calls to the patients by a clinical pharmacologist as described above.

For each patient, the following information were collected: demographic and clinical characteristics including age, sex, diagnosis (RA, PsA or AS), disease duration, current or prior use of antirheumatic medications including csDMARDs, corticosteroids and bDMARDs, comorbidities, discontinuation or switch/swap to another molecule with reason, and adverse events. Patients were considered to have discontinued treatment if they did not take that specific agent after the recommended dosing interval had passed, regardless of whether they subsequently switched to another biological agent; conversely, patients were defined “persistent” if they were still on treatment with the same drug during the last 3 months of the follow-up period. The reasons for treatment discontinuation were classified as inefficacy or AEs. Furthermore, patients were classified as switchers if they were initiated on treatment with a bDMARD other than the one reported on the index date during the study follow-up period.

Furthermore, AEs were collected through active reporting and phone calls. For each AE, the investigator (clinician or pharmacologist) recorded a detailed description of the detected AE, including the onset date, severity, time-course, duration and outcome. All AEs were coded according to the MedDRA dictionary version 20.0. SAEs were defined as events that were fatal or life threatening and resulted in a persistent or major disability or incapacity, required prolonged inpatient hospitalization, or led to a congenital anomaly or birth defect. For comparison, the number of AEs spontaneously reported for the same molecules in the same centers before starting the protocol (January 1, 2014 to December 31, 2015) were used.

### Statistical analysis

Demographic and baseline characteristics were summarized using descriptive statistics. Continuous data are presented as mean ± standard deviation (SD) or median (25–75 percentile) as appropriate, while ordinal data are expressed as number (percentage). The Fisher’s exact test for qualitative variables was used to compare the number of ADRs before and after implementing the CBPP protocol. The significance level was set at a p value < 0.05. SPSS 22.0 software (Chicago, IL) was used for statistical analysis.

## Results

### General characteristics of the study population

General characteristics of the CBPP study population are summarized in Table 1.

**Table 1:** General characteristics of the study cohorts.

	Before CBPP (n = 368)	After CBPP (n = 399)	P value
**Female sex**, n (%)	237 (64.4)	245 (61.4)	0.41
**Age**, years	56 ± 10	58 ± 11	0.02
**Follow-up**, months	11 ± 4	20 ± 2	<0.0001
**Diagnosis**			
RA, n (%)	194 (52.7)	211 (52.9)	1.00
PsA, n (%)	123 (33.4)	119 (29.8)	0.31
AS, n (%)	51 (13.8)	69 (17.3)	0.19
**Concurrent treatments**			
MTX, n (%)	97 (26.3)	137 (34.3)	0.01
LEF, n (%)	2 (0.5)	7 (1.8)	0.18
HCQ, n (%)	0	9 (2.3)	0.004
CyA, n (%)	0	6 (1.5)	0.03
CCS, n (%)	23 (6.2)	38 (9.5)	0.11
NSAIDs, n (%)	12 (3.3)	29 (7.2)	0.01
**bDMARDs prescribed**			
IFX, n (%)	58 (15.8)	49 (12.3)	0.18
ETN, n (%)	89 (24.2)	92 (23.1)	0.73
ADA, n (%)	78 (21.2)	85 (21.3)	1.00
GOL, n (%)	50 (13.6)	41 (10.3)	0.18
CZP, n (%)	5 (1.3)	14 (3.5)	0.06
ABT, n (%)	53 (14.4)	51 (12.8)	0.53
TCZ, n (%)	35 (9.51)	38 (9.5)	1.00
UST, n (%)	0	10 (2.5)	0.002
SEC, n (%)	0	19 (4.8)	<0.0001
**Adverse events**			
AEs, n (%)	42 (11.4)	125 (31.3)	<0.0001
SAEs, n (%)	0	9 (2.3)	0.004

CBPP, Calabria Biologics Pharmacovigilance Program; MTX, methotrexate; LEF, leflunomide; HCQ, hydroxychloroquine; CyA, cyclosporin A; CCS, corticosteroids; NSAIDs, Nonsteroidal anti-inflammatory drugs; IFX, infliximab; ETN, etanercept; ADA, adalimumab; GOL, golimumab; CZP, certolizumab pegol; ABT, abatacept; TCZ, tocilizumab; UST, ustekinumab; SEC, secukinumab; AEs, adverse events; SAEs, serious adverse events.

During the study period, 399 patients (245 females; mean age: 58 ± 11 years) were started on treatment with bDMARDs for active RA (n = 211, 52.9%), PsA (n = 119, 29.8%) or AS (n = 69, 17.3%) at eight rheumatology centers distributed across Calabria Region (Southern Italy). Overall, 49 (12.3%) patients received infliximab (IFX), 92 (23.1%) etanercept (ETN), 85 (21.3%) adalimumab (ADA), 41 (10.3%) golimumab (GOL), 14 (3.5%) certolizumab pegol (CZP), 51 (12.8%) abatacept (ABT), 38 (9.5%) tocilizumab (TCZ), 10 (2.5%) ustekinumab (UST) and 19 (4.8%) secukinumab (SEC); furthermore, 226 (56.6%) of them received concurrent treatment with either one or more csDMARDs, NSAIDs or corticosteroids. None received rituximab (RTX). At the time of treatment initiation, the majority (n = 287, 71.9%) of patients were bDMARDs-naïve; the remaining switched/swapped from one or more previous bDMARDs (number of previous bDMARDs range: 0–3). Data reported refer to switches during study inclusion (see Table 2 for details about switches).

**Table 2:** Details on switches between bDMARDs.

*Switch to*										
*Switch from*		**IFX**	**ETN**	**ADA**	**GOL**	**CZP**	**ABT**	**TCZ**	**UST**	**SEC**
	**IFX**		11	5(1)	8	\	2(3)	1	1	1
	**ETN**	2		13(1)	8(1)	1	5	2	2	5
	**ADA**	1	12(2)		9(1)	5	7(3)	3	3	3(1)
	**GOL**	2	3	2(1)		\	1	1(2)	1(1)	1
	**CZP**	\	\	\	\		1	1	\	1
	**ABA**	1	\	1(1)	1(1)	1		3	\	\
	**TOC**	\	2	\	1	\	3		\	\
	**UST**	\	\	1	\	\	\	\		4
	**SEC**	\	\	\	\	\	\	\	\	

Switches related to inefficacy (switches related to AEs); IFX, infliximab; ETN, etanercept; ADA, adalimumab; GOL, golimumab; CZP, certolizumab pegol; ABT, abatacept; TCZ, tocilizumab; UST, ustekinumab; SEC, secukinumab.

### Prevalence of AEs before and after starting the CBPP

**Fig 1:**
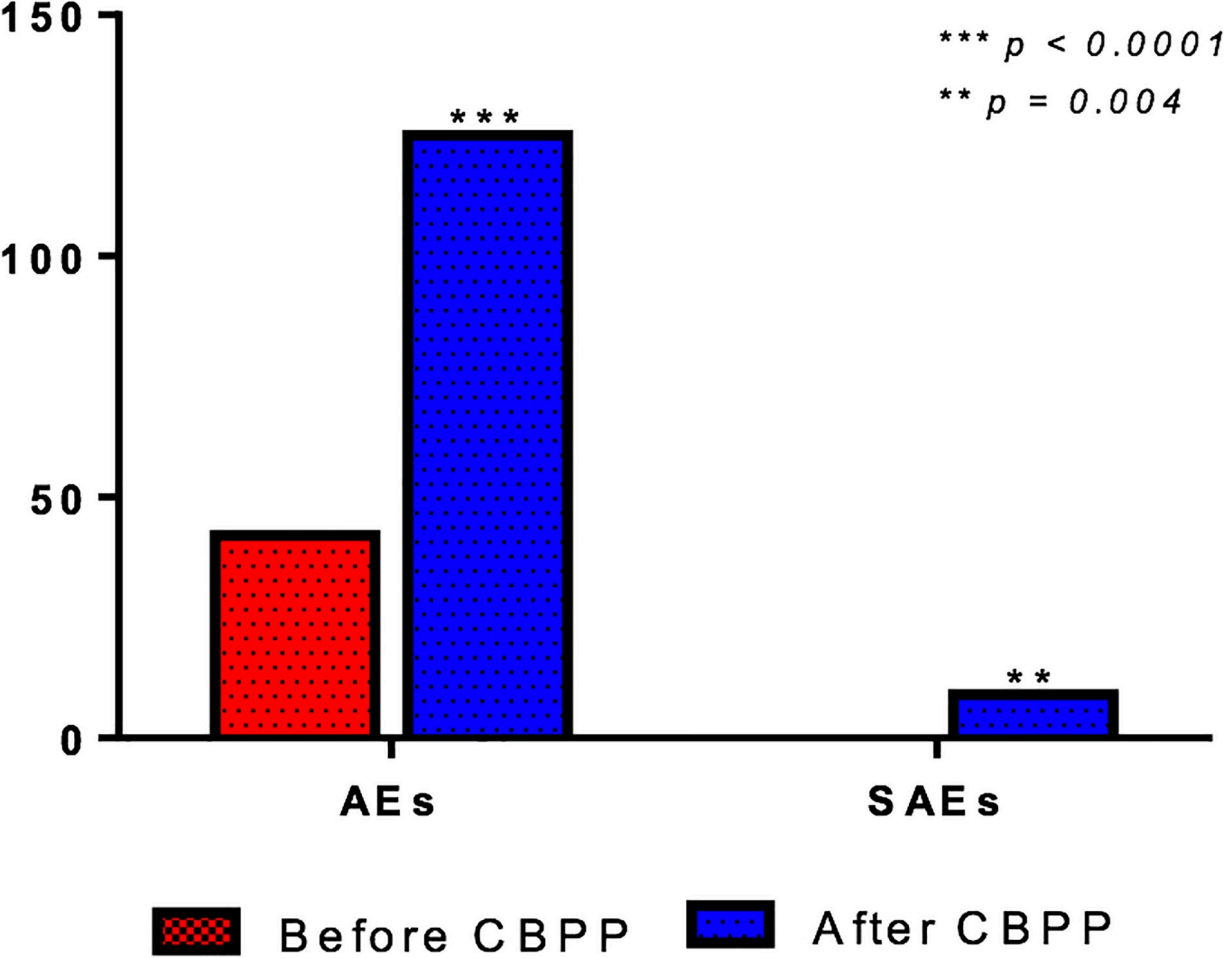
Prevalence of adverse events (AEs) and serious adverse events (SAEs) before and after CBPP protocol implementation.

As detailed in methods, the CBPP pharmacovigilance study protocol was started in January 2016. Accordingly, all patients undergoing treatment with one bDMARD at recruiting centers during the subsequent 24 months were recruited. Following the implementation of the CBPP, 125 AEs (31.3%) and 9 SAEs (2.3%) were reported during the two-years study period. In the control cohort (comprising 368 consecutive patients started on treatment with bDMARDs during a two-years period before CBPP study) only 42 (11.4%) AEs and no SAEs were reported, thus resulting in a statistically significant difference in favor of CBPP protocol (p < 0.0001, Fig 1).

**Fig 2:**
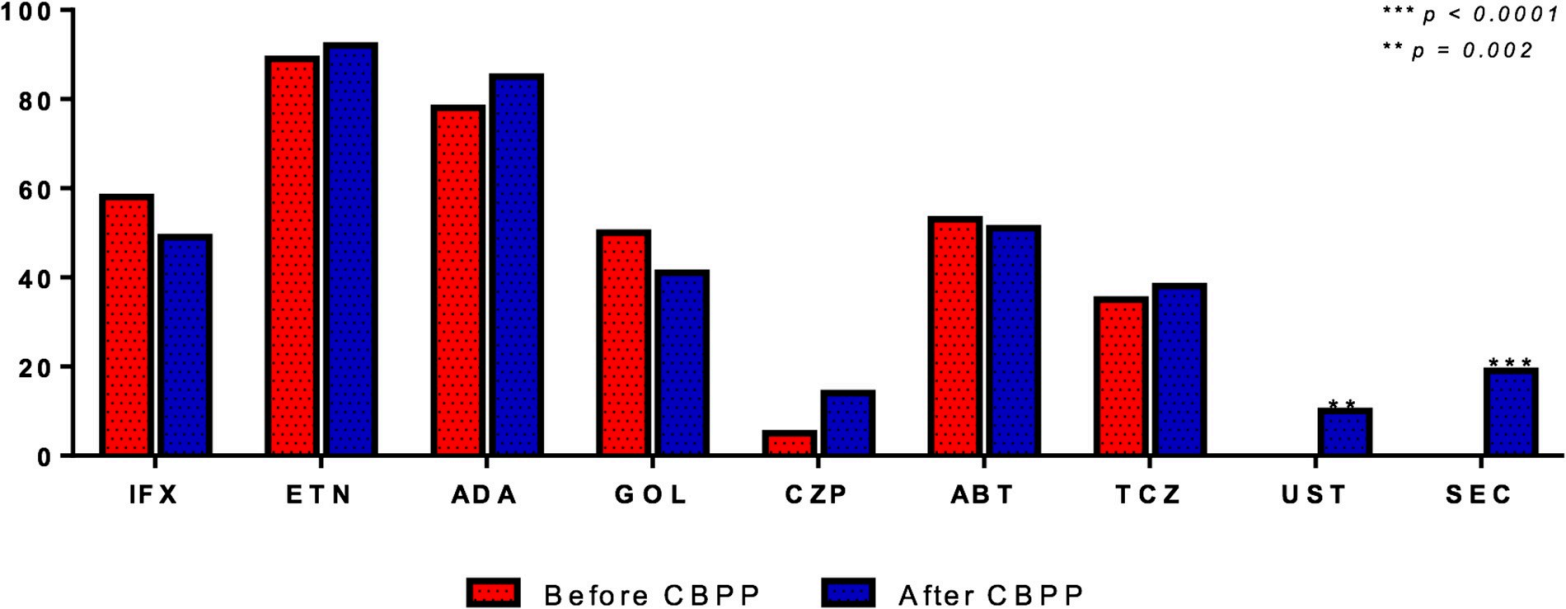
Prevalence of adverse events (AEs) before and after CBPP protocol implementation for individual bDMARDs.

In the CBPP cohort, AEs were most frequently reported with secukinumab (78.9%), golimumab (48.8%), abatacept (41.2%), certolizumab pegol (35.7%), tocilizumab (31.6%), ustekinumab (30%), etanercept (25%), adalimumab (18.8%) and infliximab (14.3%) (Fig 2).

The most common adverse events were injection site reactions and skin disorders. A detailed list of AEs classified according to the MedDRA dictionary is provided in Tables 3 and 4. A total of 9 SAEs were reported during the CBPP study period: two cases of severe uveitis leading to permanent visual loss (infliximab and golimumab), three cases thrombocytopenia leading to major bleeding (etanercept, abatacept and secukinumab), two cases of severe hemorrhage without thrombocytopenia (abatacept), one case of progressive multifocal leukoencephalopathy (adalimumab) and one case of new-onset multiple sclerosis (golimumab).

**Table 3:** MedDRA-compliant description of adverse events (AEs) after CBPP implementation.

	IFX	ETN	ADA	GOL	CZP	ABT	TCZ	UST	SEC	Total
**SOC—General disorders and administration site conditions**	**1**	**15**	**8**	**14**	**4**	**14**	**6**	**1**	**7**	**70**
PT2—Administration site reaction		15	8	14	4	13	6	1	7	68
PT3—Hyperthermia	1					1				2
**SOC—Vascular disorders**						**2**				**2**
PT—Haemorrhage						2*				2
**SOC—Skin and subcutaneous tissue disorders**	**1**	**3**	**2**	**3**		**1**	**2**	**1**	**3**	**16**
PT1—Rash	1	3	1	3		1	2	1	3	15
PT2—Nail disorder			1							1
**SOC—Ear and labyrinth disorders**						**2**				**2**
PT—Tinnitus						2				2
**SOC—Nervous system disorders**	**2**	**1**	**2**	**1**					**1**	**7**
PT—Progressive multifocal leukoencephalopathy			1*							1
PT2—Headache	2	1	1						1	5
PT3—Demyelination				1*						1
**SOC—Infections and infestations**	**1**	**1**	**2**	**2**						**6**
PT—Pneumonia viral			2							2
PT2—Herpes simplex	1	1		2						4
**SOC—Respiratory, thoracic and mediastinal disorders**	**1**	**1**	**1**				**2**			**5**
PT—Benign respiratory tract neoplasm			1							1
PT2—Dyspnoea	1	1					2			4
**SOC—Investigations**	**1**	**2**		**1**				**1**	**1**	**6**
PT—Red blood cell sedimentation rate abnormal		1							1	2
PT2—White blood cell count abnormal	1	1		1				1		4
**SOC—Blood and lymphatic system disorders**		**1**				**1**			**1**	**3**
PT—Thrombocytopenia		1*				1*			1*	3
**SOC—Gastrointestinal disorders**	**1**		**2**		**1**	**4**	**3**		**3**	**14**
PT—Nausea	1		2		1	2	2		3	11
PT2—Anal fistula							1			1
PT3—Vomiting						2				2
**SOC—Immune system disorders**	**1**			**1**						**2**
PT—Uveitis	1*			1*						2

* Classified as serious adverse event (SAE)

IFX, infliximab; ETN, etanercept; ADA, adalimumab; GOL, golimumab; CZP, certolizumab pegol; ABT, abatacept; TCZ, tocilizumab; UST, ustekinumab; SEC, secukinumab.

**Table 4:** MedDRA-compliant description of adverse events (AEs) before CBPP implementation.

	IFX	ETN	ADA	GOL	CZP	ABT	TCZ	UST	SEC	Total
**SOC—General disorders and administration site conditions**	**2**	**5**	**4**		**1**		**1**			**13**
PT2—Administration site reaction		3	2							4
PT3—Hyperthermia	1	1								2
PT4—Hyperhidrosis		1								1
PT5—Asthenia	1		2		1		1			5
**SOC—Nervous system disorders**		**2**	**1**	**1**						**4**
PT1—Headache		2								2
PT2—Demyelination				1						1
PT3—Confusional state			1							1
**SOC—Skin and subcutaneous tissue disorders**	**4**	**1**	**3**		**1**					**9**
PT1—Rash	4	1	3		1					9
**SOC—Eye disorders**			**2**							**2**
PT—Visual impairment			2							2
**SOC—Ear and labyrinth disorders**	**1**	**2**								**3**
PT1—Hypoacusis		1								1
PT2—Vertigo	1	1								2
**SOC—Gastrointestinal disorders**			**4**				**1**			**5**
PT1—Nausea			2				1			3
PT2—Vomiting			2							2
**SOC—Investigations**					**1**	**1**				**2**
PT1—Hepatitis C antibody positive						1				1
PT2—Transaminases increased					1					1
**SOC—Respiratory, thoracic and mediastinal disorders**	**4**		**1**							**5**
PT2—Dyspnoea	1									1
PT3—Throat tightness	3		1							4
**SOC—Blood and lymphatic system disorders**			**1**							**1**
PT—Thrombocytopenia			1							1

IFX, infliximab; ETN, etanercept; ADA, adalimumab; GOL, golimumab; CZP, certolizumab pegol; ABT, abatacept; TCZ, tocilizumab; UST, ustekinumab; SEC, secukinumab.

## Discussion

The therapeutic approach to inflammatory arthritides has been radically changed by the development and marketing of several bDMARDs. These molecules, differently from csDMARDs, can selectively target key pathophysiological mechanisms involved in inflammatory arthritis immunopathogenesis and, despite their significantly higher cost, represent nowadays an essential bullet in the rheumatology therapeutic armamentarium. Meta-analyses of clinical trials robustly demonstrated their efficacy and the overall favorable safety profile [22]. However, when dealing with molecules potentially affecting key physiological functions such as protective immune homeostasis, safety data produced during clinical trials may be insufficient to unravel the real impact of the treatment in clinical setting. Indeed, despite the strong methodology, clinical trials have some limitations in detecting rare or uncommon adverse events, mainly related to the restrictive inclusion criteria, the narrowly-coded disease phenotype (i.e. classification criteria) and the relatively short follow-up period. Furthermore, patients in clinical trials undergo a tight follow-up protocol allowing identification and prevention of some AEs, which is often very different from the follow-up policy used in clinical practice.

According to the World Health Organization (WHO) definition, pharmacovigilance is “*the science and activities relating to the detection*, *assessment*, *understanding and prevention of adverse effects or any other drug-related problem*”. Aim of pharmacovigilance is therefore to implement strategies to improve the early detection of AEs, to identify risk factors and special patient’s populations (i.e. elderly) and finally to produce strategies to minimize the risk thus preventing patients from being affected unnecessarily. Spontaneous reporting of suspected AEs represents the cornerstone of pharmacovigilance, allowing to rapidly detect potential warning signals related to medication use through the early detection of new AEs. Furthermore, the involvement of a wide range of healthcare professionals (physician, nurse, clinical pharmacist) confers to this epidemiological approach a role of “alarm” in the identification of events with very low frequency [23]. However, although post-marketing surveillance plays an essential role in patient safety, it is often biased by a significant degree of underreporting [24] with an overall estimate of only 6–10% of all AEs reported. This phenomenon is a major limitation of spontaneous reporting, considering that, AEs have a great impact on public health and represent a significant economic burden on both healthcare systems and society [25].

Based on these alarming data, on July 21, 2012, a new EU pharmacovigilance legislation came into force in order to strengthen and rationalize the EU pharmacovigilance system, minimize AEs and enhance drugs safety [26].

In the present study, we reported the performance of a simple, cost-effective active pharmacovigilance program in increasing the number of reported ADRs associated with bDMARDs therapy in rheumatology. Our study, by means of implementing a hybrid reporting system (stimulated spontaneous reporting and phone calls) favored a significant increase in both the number and the quality of AEs reported, in sharp contrast with the reporting behavior before protocol implementation, thus contributing to reduce the under-reporting phenomenon and providing insightful information on AEs in real life clinical practice. Following the CBPP study implementation, indeed, the number of AEs in the study cohort increased significantly (+274%) although still not reaching the figures observed in the majority of rheumatology registries such as the Italian GISEA registry [27] (AEs: 57%) or the Spanish BIOBADASER [28] (80.7%).

The crucial role of active pharmacovigilance in stimulating AEs reporting has been already demonstrated in several Italian studies including pediatric [29] and elderly patients [30]. Recently, an observational study (BIO-Cam) investigated the occurrence of AEs in naïve patients receiving biologic drugs in several settings (including rheumatology), supporting our findings that pharmacovigilance programs may represent a winning strategy to improve the knowledge of safety profile of these new drugs [31]. Similarly, the project “FarmaREL” produced a significant increase of spontaneous AEs reporting among hematology specialists [32]. Finally, an observational study conducted by an Italian National Cancer Institute confirmed the essential role of pro-active pharmacovigilance in increasing spontaneous reporting of AEs associated with anticancer targeted-therapies [33].

## Conclusions

Our study, although biased by some limitations including a relatively small sample size and the consequent lack of power for estimates of AEs for individual molecules, provides further evidence of a critical role of active pharmacovigilance in detection, reporting and analysis of AEs in rheumatology.
